# Co-twin Control Analyses Reveal Genetic Contributions to SES Influences in Mean Level and Longitudinal Change in Physical Aging

**DOI:** 10.1007/s10519-026-10256-2

**Published:** 2026-02-18

**Authors:** Deborah Finkel, Brian K. Finch, Margaret Gatz, Ida K. Karlsson, Chandra A. Reynolds, Miriam Mosing, Sneha Nimmagadda, Malin Ericsson

**Affiliations:** 1 Center for Economic and Social Research, University of Southern California, Los Angeles, CA, USA; 2 Department of Psychology, University of Southern California, Los Angeles, CA, USA; 3 Institute for Gerontology, Jönköping University, Jönköping, Sweden; 4 Department of Sociology and Spatial Sciences, University of Southern California, Los Angeles, CA, USA; 5 Department of Medical Epidemiology and Biostatistics, Karolinska Institutet, Stockholm, Sweden; 6 Institute for Behavioral Genetics, University of Colorado Boulder, Boulder, CO, USA; 7 Department of Psychology and Neuroscience, University of Colorado Boulder, Boulder, CO, USA; 8 Department of Economics, University of Southern California, Los Angeles, CA, USA; 9 Aging Research Center, Karolinska Institutet & Stockholm University, Stockholm, Sweden

**Keywords:** SES, Functional aging, Latent growth curve, Co-twin control, Gender differences

## Abstract

Socioeconomic status (SES) predicts age-related changes in health status; however, the source of SES-health associations is heavily debated. Twin studies allow tests of causal hypotheses by modeling within and between twin pair differences in longitudinal latent growth curve models (LGCM) of physical aging, examining level of functioning and rate of change with age. Three longitudinal twin studies of aging (mean age at baseline = 71.36, SD = 10.7) from the Swedish Twin Registry (*N* = 1369) included up to 27 years of follow-up on a Functional Aging Index (FAI) consisting of lung function, grip strength, walking speed, and self-report sensory functioning. SES indicators included education, financial strain, and occupation-based socioeconomic position. Pair means (between family effect) and within pair differences (within family effect) for SES were included as covariates of both intercept and slopes in a two-slope LGCM (intercept at age 75); models were corrected for sex and parental SES. LGCM results were compared across the full sample, then separately for both monozygotic and dizygotic twin pairs. Results indicated genetic confounding in the associations between multiple SES indicators and the FAI intercept. Additionally, the relationship between education longitudinal change in physical aging up to age 75 was subject to genetic confounding. These patterns were replicated among men. In contrast, findings for women pointed to shared environmental influences rather than genetic confounding, although statistical power was reduced in sex-stratified analyses. Results highlight the importance of considering the timing of socioeconomic exposures and gendered life-course trajectories when examining health inequalities in aging.

Quality of life in late adulthood is strongly influenced by the ability to maintain physical functioning and postpone dependency (Netuveli and Blane 2008; Sanders et al. 2016). Moreover, evidence suggests that functional capacity can play a critical role in offsetting the effects of disease to increase years of healthy aging (Sanders et al. 2016). The Functional Aging Index (FAI), combining gait, grip strength, lung function, and sensory ability, was developed to assess functional capacity based on methods that are minimally invasive and accessible to both practitioners and researchers (Finkel et al. 2019). FAI is strongly associated with molecular and physiological measures of biological aging (Li et al. 2020). Importantly, the FAI complements standard measures of frailty by improving prediction of outcomes such as entry into care arising from increased dependency (Finkel et al. 2019). Studies of factors that contribute to FAI suggest that loneliness (Phillips et al. 2023), subjective and objective measures of physical health (Finkel et al. 2015), and measures of socioeconomic status (SES) in both childhood and adulthood contribute to FAI in later adulthood (Finkel et al. 2024).

An extensive literature documents that SES—encompassing occupational status, income or financial strain, and educational attainment—is one of the most robust predictors of health (Mirowsky and Ross 2003). SES is associated with a broad array of outcomes including physical disability (Freedman et al. 2008), frailty (Wang and Hulme 2021), and physical functioning (Montez 2013). SES predicts change in health status over age, even after accounting for measured confounders such as environmental and biological risk factors (Elovainio et al. 2011; Marmot et al. 2013; Stringhini et al. 2012). Moreover, some evidence suggests that the impact of SES on health outcomes is amplified with age (Huxhold et al. 2017). Conversely, other studies support the “age-as-leveler” hypothesis, proposing that late-life SES convergence in health outcomes may arise from selective mortality and/or institutional equalization mechanisms, e.g., Social Security and Medicare access (Beckett 2000; Dupre 2007; Herd 2006; House et al. 1994). The robustness and breadth of these associations has led to SES being identified as a “fundamental cause” of health disparities (Freese and Lutfey 2010; Link and Phelan 1995). Nevertheless, the source of these SES-health associations continues to be heavily debated within social epidemiology and across disciplinary divides (Adler et al. 1994; Mackenbach 2012; Pappas et al. 1993).

One of the greatest challenges in observational epidemiology is drawing causal inferences from non-experimental data. Although twin designs do not solve the problem of causal inference (Bound and Solon 1999), they help strengthen the inferences that can be made by ruling out several important confounding factors. The co-twin control method provides a powerful approach for strengthening causal inference by using twins within a pair as matched controls for one another. By accounting for shared genetic and early-life environmental factors, this design allows observational cohort data to approximate key features of experimental studies (McGue et al. 2010). If an association observed at the population level remains within twin pairs after adjustment for familial factors, this suggests that the relationship is not fully explained by shared genetic or familial influences and is therefore more consistent with a causal interpretation, assuming other relevant confounders have been adequately addressed. In constrast,, the failure to find that twins who differ in SES also differ in health outcomes would imply the association is not causal but rather due to genetic and shared environmental confounding. Past studies have used the co-twin control design (i.e., the study of twins from pairs discordant on SES) to test the association of SES with various health outcomes (McGue et al. 2010; Rutter 2009). While most of these studies have found that the association of attained SES with health outcomes is substantially attenuated or eliminated within twin pairs discordant on SES (Amin et al. 2015; Behrman et al. 2011; Ericsson et al. 2024; Madsen et al. 2014; Walters et al. 2025), some studies report significant within-pair effects (e.g., (Gerdtham et al. 2016; Lundborg 2008).

Education, occupation, or income are often used independently as indicators of SES, or they may be combined in a single measure; however, it is possible that mechanisms of their impact of health outcomes are distinct (Link and Phelan 1995). While education commonly precedes and informs occupational attainment, the relationship is not strictly chronological. Education tends to stabilize earlier in life, whereas occupation and occupational status reflect dynamic conditions across adulthood that may exert more immediate effects on aging outcomes (Herd et al. 2019; Ross and Wu 1996). Moreover, cumulative advantage theory posits that education represents a form of human capital, reflected in the capacity to acquire and effectively use health-related information (Herd et al. 2019; Lynch 2003, 2006; Ross and Wu 1996). As a result, SES differences in health status grow with increasing age, arising both from amplification of early differences and continued accumulation of health benefits for the already advantaged (Mirowsky and Ross 2003). From a life-course perspective, early stages of aging may represent periods during which socioeconomic advantages are more strongly expressed, as higher SES individuals are better positioned to accumulate health-promoting resources that support functional capacity. These early advantages may be amplified over time, resulting in increasing differences in functional aging during the earlier phases of aging. In contrast, the age-as-leveler hypothesis suggests that at later ages the aging process itself overwhelms the education advantage, resulting in reduction in education disparities in health in late adulthood (e.g., (Herd 2006; House et al. 1994). At more advanced ages, functional aging may be increasingly shaped by biological constraints, reducing the relative importance of socioeconomic resources. As functional limitations become more prevalent, the ability of education and other SES indicators to buffer age-related decline may decrease, consistent with the age-as-leveler hypothesis. For example, examinations of measures of functional aging have identified different covariates of longitudinal change before and after age 75, with SES playing a larger role before age 75 (Finkel et al. 2015; Sternäng et al. 2015).

The International Social Economic Index (ISEI) was designed to capture the socioeconomic position associated with occupations by creating a continuous, cross-nationally harmonized measure of occupational status (Ganzeboom and Treiman 1996). This measure captures how occupations mediate between education and income, and may reflect work context, prestige, or structural constraints not fully captured by education or earnings individually. ISEI maximizes the indirect association between education and income through occupation and is an “optimal score” that best represents how education is translated into earnings through occupational placement (Hauser and Warren 1997). ISEI shows incremental predictive validity for health outcomes over and above education measures (Volkers et al. 2007), although other studies report that high occupational status cannot compensate for lower education (Nocon et al. 2007). Moreover, the social and health implications of occupation may depend not only on objective job characteristics, but also on subjective perceptions of financial strain and economic security (Glei et al. 2018). For example, occupation and income may not provide a complete picture of purchasing power or wealth, and the same level of income may reflect more or less financial strain in different cost-of-living contexts (Cundiff and Matthews 2017). Previous studies have demonstrated that subjective measures of financial strain predict physical health (Singh-Manoux et al. 2005), even after controlling for income and education.

Thus, our understanding of the etiology of associations between SES and physical functioning can be expanded by considering component measures of SES (e.g., education, occupation-based socioeconomic position, and financial strain) independently as they tap different aspects of environmental context and individual experiences. They may also reflect different portions of the lifespan, with education completed in young adulthood, occupation-based socioeconomic position likely peaking in midlife, and financial strain reflecting current conditions. Socioeconomic conditions likely hold different meanings and implications across generations and birth cohorts. Historically, access to education was limited, which consequently shaped other socioeconomic opportunities and life trajectories. In Sweden, today characterized by a universal welfare system, earlier generations experienced substantial inequalities in educational access, particularly among women and individuals from lower social classes (Erikson and Jonsson 1996). Sweden provides a particularly informative context for examining socioeconomic influences on functional aging, given the substantial expansion of the welfare state, major educational reforms, and the growth of public-sector employment during the mid-20th century (Sundin and Willner 2007). These structural changes altered the distribution and meaning of socioeconomic resources across the life course, particularly for cohorts born earlier in the past century. Within this historical setting, women experienced more limited access to education and occupational advancement than men, while subsequent policy reforms disproportionately improved women’s educational attainment and labor market participation (Bjorklund and Palme 2000; Fischer et al. 2019; Bhalotra et al. 2022). These cohort- and sex-specific differences in socioeconomic opportunities motivate an explicit examination of whether associations between socioeconomic status and functional aging vary by both life-course timing and sex.

Aims of the current analysis were first, to investigate the impact of three measures of SES on mean level and longitudinal changes in trajectories of functional aging. Previous investigations indicated different rates of change in FAI before and after age 75; therefore, we predict that the association of SES measures with longitudinal change will differ for mean level and early vs. later aging. The innovation of the second goal is the integration of the co-twin control approach with latent growth curve models to test the quasi-causal effects of each measure of SES on functional aging to examine familial confounding versus causal interpretations. Again, we predict that the mechanisms of relationships between exposures (SES) and FAI will vary for mean level and early vs. later aging. Third, given sex differences in access to socioeconomic opportunities in these cohorts, we examined sex differences in the mechanisms of the associations between SES and FAI.

## Method

### Participants

Data came from three studies that are part of the Interplay of Genes and Environment across Multiple Studies (IGEMS) consortium (Finkel et al. 2025): Swedish Adoption/Twin Study of Aging (SATSA; Finkel and Pedersen 2004), Origins of Variance in the Oldest Old (OCTO-Twin; McClearn et al. 1997), and Aging in Women and Men: A Longitudinal Study of Gender Differences in Health Behavior and Health among the Elderly (GENDER; Gold et al. 2002). Recruitment and testing procedures for the three studies have been described previously. In brief, all three samples were drawn independently from the population-based Swedish Twin Registry (Lichtenstein et al. 2002), resulting in non-overlapping samples that were evaluated utilizing similar protocols. SATSA and OCTO-Twin recruited only same-sex pairs and OCTO-Twin focused on twins in their 80 s, whereas GENDER recruited only opposite-sex pairs in their 70s. In-person testing (IPT) took place in locations convenient to participants, such as district nurses’ offices, health-care schools, long-term care clinics, or at the participant’s home. IPT intervals across the studies ranged from two to four years. Socioeconomic status and functional aging variables were available from at least one testing occasion for 1369 individuals: 555 from SATSA, 378 from OCTO-Twin, and 436 from GENDER. Including parental occupation in some models resulted in a slightly reduced sample size (*N* = 1293). All participants were of European ancestry and 51.89% of the sample was female. Three or more waves of data were available for 58% of the sample. As shown in Table 1, the sample ranged in age from 42 to 93 years at baseline and mean age at baseline was 71.36 (*SD* = 10.69). Follow-up ranged from 0 to 27 years, with a mean of 10.20 years (*SD* = 7.28).

Original IRB approval for the SATSA, OCTO-Twin, and GENDER studies was obtained at Karolinska Institutet (document numbers 84:61, 98:380, and 93:226, respectively). Both SATSA and GENDER have updated approvals by the Regional Ethics Review Board in Sweden (document numbers 2010/657 – 31/3 and 2021–02095, respectively).

### Measures

#### Socioeconomic Status (SES)

Four measures of SES were used in the current analyses. *Education* was categorized into 9 levels using the International Standard Classification for Education (ISCED; UNESCO 2012). Values range from 0 (no education or less than primary education) to 8 (doctoral degree or equivalent). *Occupation-based socioeconomic position* was rated based on the International Socio-Economic Index of major lifetime occupation (ISEI; Ganzeboom and Treiman 2003). The ISEI combines information about education, occupation, and income to indicate the status of the occupation. Values can range from 15 to 89 and higher scores indicate higher prestige. *Parental occupation* was coded according to the Swedish Socioeconomic Index (SEI; SCB, 1989) which classifies the major lifetime occupation reported for parents into a scale ranging from 1 (unskilled worker) to 4 (professionals). Owing to missing data, the analytic sample for parental occupation was limited to 1,293 individuals (see Table 1). Parental occupation was coded as a household measure and was based on the parent with the higher occupational level. *Financial Strain* (FS) at baseline was harmonized as part of the IGEMS consortium (Finkel et al. 2022) and consists of four items that were common across most of the studies: how well does your money cover your needs, do you have difficulty covering your monthly expenses, how does your economic situation compare to others of the same age, and do you usually have enough money for extra treats. Factor analysis supported a single financial strain measure, and factors scores were generated on available items for each individual using factor loadings calculated in full IGEMS sample. Factor scores were then translated to T-score metric (mean of 50 and SD of 10 within each sample), with higher scores indicating more financial strain.

#### Functional Aging Index (FAI)

FAI was designed to complement existing measures of biological aging and frailty indexes by focusing on functional capacity (Finkel et al. 2019). It includes measures of 4 functional biomarkers: self-reported sensory abilities (vision and hearing), peak expiratory lung flow, grip strength, and gait speed. Before calculation of FAI, grip strength was regression-adjusted for sex and peak expiratory flow was adjusted for body volume through dividing it by the individual’s squared height in meters. The four variables were standardized separately based on baseline means and standard deviations, and grip strength and peak expiratory flow were reverse scored to ensure that higher scores indicated poorer performance. Finally, the composite score was translated to the T-score metric; higher scores on FAI indicate poorer functioning.

### Statistical Method

Due to the range in age at each wave, an age-based *latent growth curve model* (LGCM) was used to estimate trajectories of change with age in FAI. The structural model can be considered as a multi-level random coefficients model (Bryk and Raudenbush 1992; McArdle and Anderson 1990). The model estimates both fixed effects—population-level parameters representing the average growth trajectory across the sample—and random effects, which capture individual variability in rates and patterns of change around that average. The age basis reflects each participant’s age at measurement, centered at 75 years (i.e., observed age minus 75).The age basis serves as a marker for the age of the subject at each time of measurement, adjusted for the centering age. Therefore, age basis coefficients are defined as an individual’s observed age at each measurement occasion minus the centering age (75 years), divided by 10 to support parameter estimation. Accelerating change with age in FAI is best captured using a two-slope model: slope 1 estimates the rate of change up to age 75 and slope 2 estimates the rate of change after age 75 (Finkel et al. 2019). Sex and parental SEI were included as covariates as appropriate; previous analyses indicated sex differences in the intercept of the LGCM model for FAI, but not in the slope estimates (Finkel et al. 2017).

**Fig. 1:**
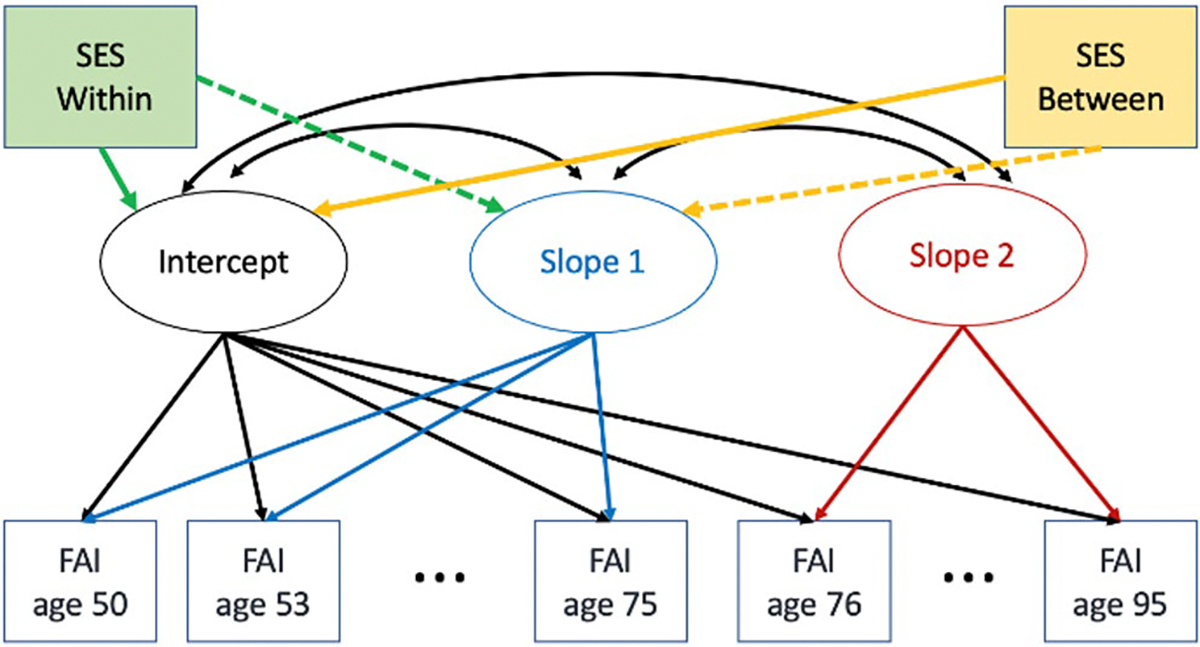
Latent growth curve model indicating spline function with intercept at age 75 and SES within pair and SES between pair as covariates of the intercept and slope 1 (as appropriate). None of the SES measures were related to slope 2

First, in *phenotypic analyses*, the relationship between the individual SES variables (ISCED, ISEI, FS) and FAI trajectory were ascertained by including each SES variable as a covariate of the LGCM parameters: intercept, slope 1, and slope 2. Each SES variable was tested separately. Second, *co-twin control analyses* (Kendler et al. 1993; McGue et al. 2010) were used to explore potential genetic and shared environmental mediation of the identified associations between the SES variables and the LGCM parameters for FAI. The approach assumes that monozygotic (MZ) twins share 100% of their genes and dizygotic (DZ) twins share on average 50% of their segregating genes. Shared environmental influences contribute to similarity within families, and unique environmental influences contribute to differences within families. The between-within pair covariate model can test the hypothesis that the twin with higher values on the SES variable will have a lower (better) intercept on FAI and slower rates of increase, statistically adjusting for familial genetic and shared environmental factors that make twins similar (Mosing et al. 2016). Between-pair differences were indicated by calculating the pair mean on the SES variable. Within-pair differences were calculated by subtracting the between-pair mean from the SES value for each member of the pair. Within and between pair variables were then incorporated in the LGCM as covariates of the intercept and slopes, as appropriate based on phenotypic analyses (see Fig. 1). Third, possible *sex differences* in the association of between-pair means and within-pair differences on SES variables with LGCM parameters for FAI were also examined.

If the SES variable, per se, contributes to FAI aging trajectories, then the association of within-pair differences and the LGCM parameter will exist not only at the population level, but also within MZ and DZ pairs discordant for the SES variable, i.e., the strength of the relationship of the within-pair variable with the LGCM parameters will not be attenuated by the degree of relatedness (see Supplemental Fig. 1). If, however, the relationship is not directly causal but arises from genetic factors common to both the SES variable and FAI, then the relationship of the within-pair variable with the LGCM parameters will be attenuated (or absent) for MZ twins but less so for DZ twins. If the relationship arises from shared environmental factors common to both the SES variable and FAI, then the relationship of the within-pair variable with the LGCM parameters will be attenuated equally for MZ and DZ twins, with no impact from their degree of relatedness.

The random and fixed effects parameter estimates were obtained using PROC Mixed in SAS 9.4 and phenotypic models were corrected for twinness by modeling both between and within pair variance in the random effects. Nested LGCM were compared using a likelihood ratio test (LRT), which is the difference in the model fit statistic (log likelihood) for the two models, with degrees of freedom equal to the difference in parameters estimated.

## Results

### Descriptive Statistics

Descriptive statistics for the three twin studies and the combined sample are presented in Table 1. Descriptive statistics for men and women are presented in Supplemental Table 1: differences between men and women were statistically significant for only 4 variables. Women has significantly longer mean follow-up than men (10.73 vs. 9.62 years) and significantly higher mean parental SEI and men had significantly higher mean ISCED and ISEI; but there was no significant sex difference in mean FS. Estimated longitudinal trajectories of FAI, calculated separately for men and women, are presented in Supplemental Fig. 2. Women had higher mean level FAI, and in both groups slope 2 estimates (after age 75) were twice as large as slope 1 estimates. Correlations among FAI and the SES measures are presented in Table 2 for the whole sample, separately for men and women, and also for different age ranges (before and after age 75), and three birth cohorts of approximately equal size (1900–1914, 1915–1923, 1924–1948). Correlations were strongest between ISCED and ISEI in all groups, and there was evidence that this correlation tended to increase from early (0.55) to later (0.67) birth cohorts (z = 2.94, *p* <.01). FS was more modestly correlated with ISCED and ISEI. The correlation between FS and ISEI was significantly stronger in women than in men (z = 2.06, *p* <.05) and tended to decline from early to later birth cohorts (z = 2.33, *p* <.05). Although trends were not significant, correlations between FAI and ISCED and FAI and ISEI also increased from early to later birth cohorts. MZ and DZ twin correlations, respectively, for the 3 SES variables were 0.53 and 0.43 (ISCED), 0.53 and 0.29 (ISEI), and 0.46 and 0.11 (FS). Twin correlations are reported separately for men and women and Supplemental Table 1.

### Phenotypic Analyses

The base LGCM for FAI, corrected for sex and parental SEI, was compared to models incorporating each SES variable, to determine whether the variable impacted the longitudinal trajectory for FAI. Models with and without the SES variables are nested, so the LRT was used to test for significant change in model fit when SES variables were added. In each case, adding the SES variable resulted in a significant improvement in model fit: LRT (df = 3) was 45.3 for FS (*p* <.01), 8.3 for ISEI (*p* <.05), and 47.5 for ISCED (*p* <.01). Table 3 presents the parameter estimates for the models including the interaction of intercept, slope 1, and slope 2 with each SES variable (note that slope parameters estimate change over 10 years). As expected, sex was associated with the intercept of the LGCM for FAI. Parental SEI was only associated with FS, so parental SEI was not included in co-twin control models of ISEI and ISCED, in order to maximize sample size. Both slopes were significantly greater than zero and slope 2 was approximately twice as large as slope 1, indicating accelerating change in FAI after age 75. All three SES variables were significantly related to the intercept of the LGCM, such that higher FS was associated with higher FAI (poorer functioning), and higher ISEI and ISCED were associated with better functioning. ISCED was also significantly related to slope 1: higher ISCED was associated with a slower rate of functional loss up to age 75. Finally, 95% CIs for SES effects on slope 2 were narrow and centered near zero, suggesting little to no SES differentiation in functional decline after age 75.

### Co-twin Control Analyses

Co-twin control analyses focused on the association among between-pair means and within-pair differences on the SES variables and the intercept (all 3 SES variables) and slope (only ISCED) of the LGCM. The models were run on all complete pairs, and then separately for MZ and DZ pairs to examine the relative attenuation of associations when familial relationships are considered. Table 4 presents the covariate terms for the between and within-pair values for each SES variable associated with the intercept or slope 1, as appropriate. A complete list of parameter estimates is reported in Supplemental Table 2. Based on the phenotypic results, parental SEI was included in the model for FS, only. Between-pair parameters for the impact of FS on the intercept of the FAI were all about 0.20 and significantly different from zero. Within-pair estimates demonstrated some attenuation of the relationship, but the values in the full sample and the DZ sample are still significantly different from zero. In contrast, within-pair estimates for MZ twins did not differ significantly from zero. Moreover, the MZ within-pair estimate was significantly less than the DZ within-pair estimate (*t*(df = 4057) = 2.32, *p* <.05). This pattern of attenuation suggests genetic confounding of the relationship between FS and the intercept of the LGCM for FAI. In contrast, results for ISEI parameters indicated significant associations of between-pair means with the intercept, but little or no attenuation of this relationship in the within-pair difference parameters in any of the models (full sample, MZ, and DZ) and no significant difference between MZ and DZ within-pair estimates. Point estimates for within-pair effects were similar in magnitude for MZ and DZ twins, but the MZ estimate was non-significant with wide confidence intervals, so evidence for a strictly causal interpretation is suggestive rather than definitive.

The impact of ISCED on both the intercept and slope 1 of the LGCM model for.

FAI was examined in a co-twin control model. The results for the intercept are similar to the results for FS: between-pair parameters were significant in all three models (full, MZ, and DZ samples) and within-pair parameters were significant and showed no attenuation in the full and DZ sample. In fact, the within-pair estimates in the full and DZ samples were somewhat larger than the between-pair estimates, likely a result of unique environmental influences and/or error variance. The within-pair parameter in the MZ sample was not significantly different from zero. This pattern of attenuation in the MZ sample, only, is consistent with genetic confounding of the relationship between ISCED and the intercept of the LGCM for FAI. The pattern of results for slope 1 is similar to the results for the intercept. In both the full and DZ samples, the within-pair estimate is larger than the between-pair estimate, whereas in the MZ sample the within-pair estimate shows attenuation from the between-pair estimate. Again, the results in the full and DZ sample suggest a role for unique environmental influences and/or error variance, whereas the MZ results suggest genetic confounding of the relationship between ISCED and slope 1 (change with age up to age 75) in the LGCM for FAI.

### Sex Differences

**Fig. 2:**
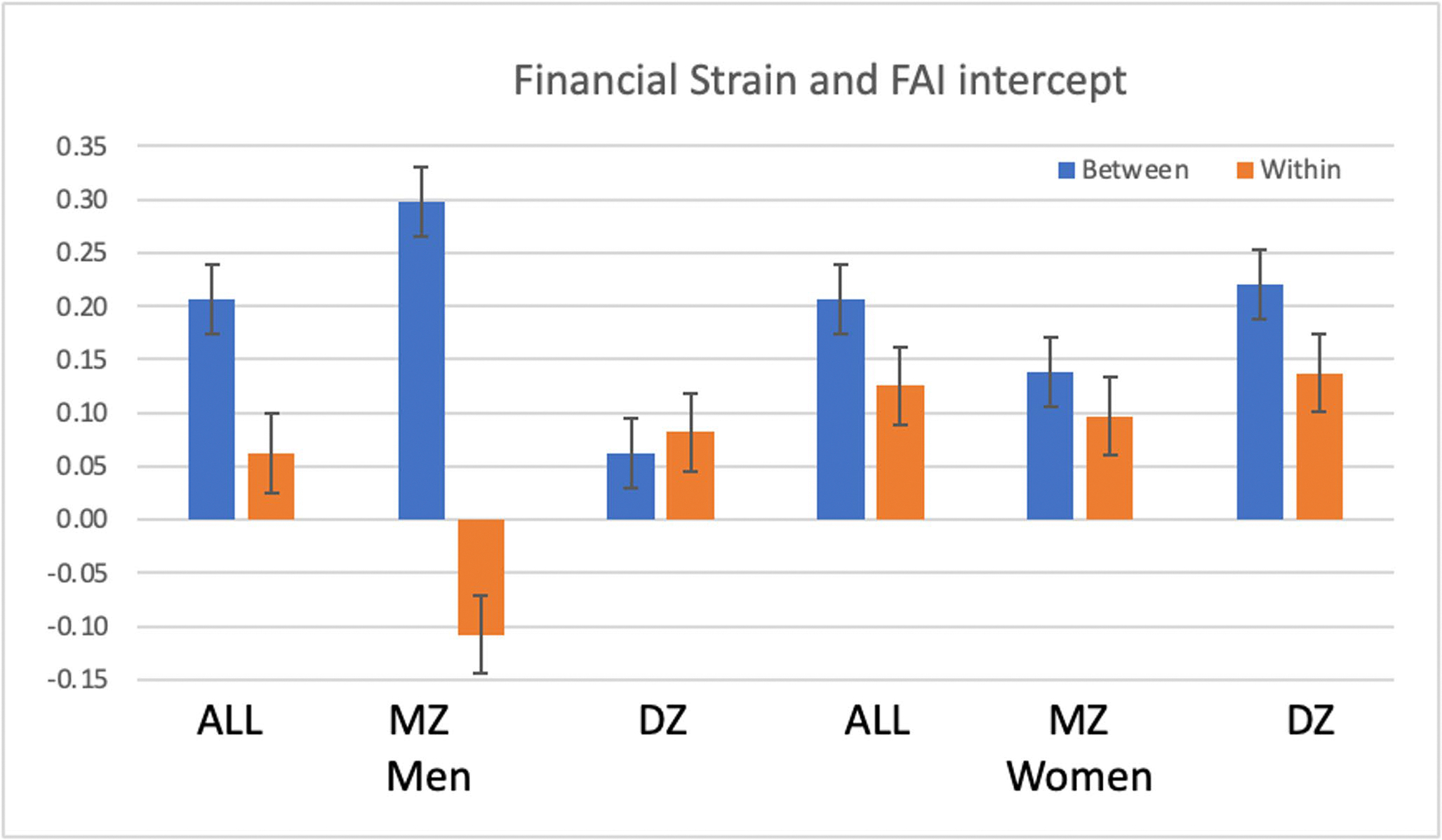
Parameters estimates for the association of within twin pair and between twin pair variance in Financial Strain with the intercept of the intercept of the latent growth curve model for FAI, estimated for the full sample, MZ twins, and DZ twins, separately for men and women

In the final step of the analyses, sex differences in the results were tested by adding interaction terms between sex and the within and between pair effects in the LGCM for the intercept, only, as phenotypic modeling indicated no sex differences in slope estimates. A complete list of parameter estimates is reported in Supplemental Table 3. A base model was estimated for women, and then the extent of adjustment in each parameter was estimated for men; these estimates are presented in Table 5. There were no significant sex differences in the pattern of results for ISEI: the pattern of results consistent with a causal model was equally true for males and females. For FS, significant sex differences were found in estimates of both within and between twin effects in the MZ sample: larger between-pair estimate and smaller within-pair estimate for men (see Fig. 2). As a result, significant attenuation of the effect of FS on FAI was found in male MZ twins, but in female MZ twins attenuation of the FS effect on FAI was minimal. Thus, results indicated genetic confounding of the relationship between FS and FAI for men but the pattern was suggestive of shared environmental confounding of FS on FAI for women. Sex differences for the effect of ISCED on intercept of FAI indicate a complex pattern of differences across groups. Similar to the results for FS on FAI intercept, the results for ISCED on FAI intercept indicate attenuation of the within-pair estimate in male MZ twins, but in female twins attenuation of the within-pair estimate was similar for MZ and DZ twins. Thus, results indicated familial confounding for both men and women, although the results were suggestive of genetic confounding of the relationship between ISCED and FAI for men but suggestive of shared environmental confounding of ISCED on FAI for women.

## Discussion

In the present study, we investigated the influence of three indicators of socioeconomic status (education, occupation-based socioeconomic position, and financial strain) on functional aging, both in terms of mean level differences and longitudinal trajectories before and after age 75. By studying a cohort of twins, we further examined whether these associations were confounded by familial (genetic or shared environmental) influences. In the initial phenotypic analyses, we could confirm previous findings (Finkel et al. 2015, 2024), showing that higher SES was associated with better outcomes on the Functional Aging Index (FAI) at the mean level. Specifically, higher education, higher SES, and lower financial strain were related to a more favorable FAI. However, when analyzing socioeconomic influences on trajectories of change in FAI, only education showed a statistically significant association with change before age 75, and none of the predictors were associated with change after age 75. These results suggest that socioeconomic differences in functional aging are more evident at younger old ages, consistent with cumulative advantage processes across the life course. In later life, however, these differences appear to attenuate, indicating that advancing age and biological vulnerability may reduce the protective effects of socioeconomic resources on functional aging. After adjusting for familial confounding, the associations for education and occupation-based socioeconomic position remained, suggesting a potential causal effect of these SES indicators on functional aging. In contrast, the within-pair estimates for financial strain were strongly attenuated. When we stratified the models by zygosity, the effects of education and financial strain on FAI could largely be attributed to genetic confounding, rather than shared environmental influences. For occupation-based socioeconomic position, however, there was no further attenuation in either dizygotic (DZ) or monozygotic (MZ) twin pairs, supporting a more direct causal impact of occupation-based socioeconomic position on FAI.

Although many genetic and environmental factors contribute to occupation, occupation-based socioeconomic position still appears to have a causal impact on physical aging. This association may reflect the physical demands of different occupations, which are likely to contribute directly to differences in physical function in later life. Alternatively, higher socioeconomic position may be associated with better access to resources that support health. The genetic confounding observed for education and financial strain may reflect shared genetic factors that influence both educational attainment and health-related behaviors, or genetic overlap with traits linked to physical health and aging. A similar interpretation may apply to the genetic component of perceived financial strain, which is a subjective measure that may also capture personality-related traits.

Education, occupation, and wealth are known to be genetically correlated (Liu 2019). This implies that pathways to FAI are unlikely to be completely independent and that the associations among SES indicators may themselves be biased by genetic confounding. Moreover, the three different socioeconomic indicators examined in this study likely capture different life-course periods: attained education is usually completed in early adulthood (especially for the cohorts in our sample) and may reflect early life/familial advantages, occupation-based socioeconomic position represents adulthood and midlife and captures social and environmental exposures in adulthood, and financial strain, measured at baseline, may reflect a later period in life. Accounting for these temporal dimensions elucidates how socioeconomic conditions operating across the life span accumulate to influence functional aging. In our data, education and occupation-based socioeconomic position were strongly correlated, whereas both were only modestly correlated with financial strain. This aligns with prior research showing that income is strongly associated with late-life health, independently of other SES factors (Darin-Mattsson et al. 2017).

We observed no meaningful sex differences in the correlations between education and occupation-based socioeconomic position, or between education and financial strain. However, the correlation between occupation-based socioeconomic position and financial strain was notably more negative among women. Because socioeconomic circumstances differed markedly between men and women in these cohorts, we further examined whether associations between SES and FAI varied by sex. These analyses were restricted to intercepts, as the phenotypic results indicated no sex differences in FAI change over time.

The results suggested that the degree of familial and genetic confounding differed by SES indicator. For occupation-based socioeconomic position, there was no evidence of sex differences, and the results for both men and women were similar to those in the total sample, supporting a causal influence of socio-economic position (ISEI) on FAI at age 75. For education, results were less conclusive, we found evidence of familial confounding in both sexes, with indications that this was primarily genetic among men and shared environmental for women. For financial strain, sex differences emerged both between and within twin pairs. Among men, the association between financial strain and FAI appeared genetically confounded, whereas among women no familial confounding was detected, in line with a potentially causal association. However, sample sizes were more limited in the sex-stratified analyses, which may have reduced statistical power, accordingly, these findings should be interpreted with appropriate caution.

Given the historically reduced opportunities for education and occupational advancement among women born between 1900 and 1948, it seems reasonable that the results indicate the link between SES and physical aging in women may reflect environmental rather than genetically influenced factors, highlighting that when opportunities are constrained, environmental conditions may overshadow genetic potentials in shaping aging trajectories. Indeed, our data showed increasing correlations between occupation-based socioeconomic position and education across cohorts, which may be related to welfare state expansion beginning in the mid-20th century, particularly policies benefiting women (Bhalotra et al. 2022; Bjorklund and Palme 2000). Educational reforms and the expansion of public-sector employment likely promoted women’s labor market participation (Fischer et al. 2019) and reduced gender gaps in earnings. Bjorklund and Palme (2000) reported that the rise in women’s labor market participation narrowed income inequalities within and between families, an effect further strengthened by welfare expansion.

Although financial strain was similarly associated with functional aging in both sexes, the underlying mechanisms may differ. Among men, the link may reflect genetic influences on education, occupation-based socioeconomic position, and financial traits that also affect health. Among women, the association may be more strongly shaped by social and contextual factors, and therefore less genetically determined. Supporting this interpretation, Finnish research found that lifetime earnings are substantially heritable—more so for men than women—and largely independent of education (Hyytinen et al. 2019).

Risk factors for aging and health evolve across the life course. For instance, a physically demanding job may have lasting health effects, but once retired, such strain is no longer cumulative. Similarly, income tends to equalize in later life, which may reduce socioeconomic disparities in health. In such more equal environments, genetic influences often become more pronounced, a phenomenon consistent with the concept that heritability increases when environmental variation decreases (Tucker-Drob and Bates 2016). It could even be argued that the environmental context differs by sex, men may experience a more equalized environment than women, potentially amplifying heritability estimates.

Finally, Sweden’s universal healthcare system, largely independent of income or SES, likely attenuates late-life inequalities in functional aging. As women generally exhibit better care-seeking behavior, this may partly explain their longer life expectancy.

### Strengths and Limitations

This study has several notable strengths. First, it draws on three subsamples from the population-based Swedish Twin Registry, utilizing rich longitudinal data that includes in-person testing and both current and retrospective measures of socioeconomic conditions. These data enabled us to assess socioeconomic influences across the adult life course, in relation to functional aging. A key strength is the use of a family-based twin design, which allowed us to not only examine associations between SES and functional aging (FAI), but also adjust for unmeasured familial confounding, including shared environmental and genetic influences. This is a major advantage over conventional observational studies and enhances the causal interpretability of our findings.

However, several limitations should be acknowledged. One primary limitation is the relatively small sample size, particularly for within-pair analyses that require complete twin pairs and further stratification by zygosity. The smaller number of monozygotic (MZ) twin pairs compared to dizygotic (DZ) pairs reduced statistical power for detecting within-pair effects, especially for sex-stratified analyses. For instance, the GENDER sample, composed exclusively of opposite-sex DZ twins, could not contribute to MZ estimates. As with all aging studies, our results are subject to selection biases related to health and mortality. Healthier individuals are more likely to survive into older ages and participate in studies, which may attenuate associations and affect generalizability. This process often described as mortality selection interacts with the concept of cumulative disadvantage and may also contribute to age-as-a-leveler effects, where SES-related differences in outcomes become less pronounced in advanced age (Dupre 2007). This survival bias may have influenced the observed association between SES and FAI. Rehnberg (2020) found that the link between income and mortality was strongest among individuals in good health, while those in poor health showed weaker income-mortality associations. This suggests that health status may be a more relevant predictor of mortality than age in late life. Similarly, Fors et al. (2021) reported no improvements in life expectancy for women with the lowest incomes, highlighting persistent SES inequalities in longevity in Sweden. In addition, it should be noted that the ISEI measure has certain limitations. It does not reflect occupation per se; rather, the purpose of the scale is to capture a weighted combination of education and income. Moreover, the measure was initially developed using data from men only. Nevertheless, ISEI has been widely used in a large number of studies and can therefore enhance comparability with previous research (Oesch et al. 2025). Finally, limitations inherent to the co-twin control design should be considered. These include potential measurement error, reduced variability in within-pair differences, and the degree of correlation between twins in both predictors and confounders. When within-pair correlations are low or measurement error is high, estimates may be biased or underpowered (Frisell et al. 2012; Gustavson et al. 2024).

## Conclusion

This study provides evidence that socioeconomic factors across the life course are associated with functional aging in later life. By leveraging a twin design, we were able to account for familial and genetic confounding, supporting a potential causal role for occupation-based socioeconomic position and, to some extent, education. In contrast, the association between financial strain and functional aging appeared more genetically confounded among men, but not among women, suggesting that social and contextual factors may play a greater role for women. These findings underscore the importance of considering both the timing of socioeconomic exposures and gendered life-course trajectories when examining health inequalities in aging.

## Supplementary Material

Supplemental Material

**Supplementary Information** The online version contains supplementary material available at https://doi.org/10.1007/s10519-026-10256-2.

## Figures and Tables

**Table 1:** Sample demographics

Variable	SATSA	OCTO-Twin	Gender	Full sample

N individuals	555	378	436	1369
N individuals with parental SEI	537	320	436	1293
N of MZ/N of DZ pairs	75/122	67/59	0/190	142/371
MZ/DZ pairs with parental SEI	73/117	50/49	0/190	123/356
Percent female	55.17	56.08	46.79	52.68
Birth year range	1903–1948	1900–1913	1916–1925	1900–1948
Age range at baseline (yrs)	42–83	79–93	70–81	42–93
Mean age at baseline (SD)	61.12 (8.22)	82.99 (2.65)	74.32 (2.61)	71.36 (10.69)
Mean waves participation (SD)	4.42 (2.37)	2.73 (1.50)	2.26 (0.85)	3.27 (2.02)
Mean length follow-up (SD)	16.30 (7.73)	5.37 (2.70)	6.61 (1.92)	10.20 (7.28)
Mean ISCED (SD)	2.06 (1.55)	1.37 (0.99)	1.37 (0.91)	1.81 (1.39)
Mean financial strain (SD)	51.66 (9.97)	44.14 (10.92)	46.96 (10.48)	48.09 (10.86)
Mean ISEI (SD)	39.53 (19.32)	31.79 (18.07)	36.01 (19.15)	36.27(19.17)
Mean parental SEI (SD)^a^	2.09 (0.91)	2.04 (0.92)	2.18 (0.85)	2.11 (0.89)

*MZ* monozygotic twin, *DZ* dizygotic twin, *ISCED* international standard classification of education, *ISEI* international socio-economic index, *SEI* socio-economic index

**Table 2:** Correlations among FAI and SES measures

Sample	Correlations among SES measures			Correlations of FAI at baseline with SES measures		
	ISCED × ISEI	ISCED × FS	ISEI × FS	ISCED	FS	ISEI

Total sample	0.62	−0.11	−0.16	−0.28	0.07	−0.19
Men	0.64	−0.09	−0.10	−0.24	0.09^a^	−0.17
Women	0.60	−0.12	−0.21	−0.28	0.03^a^	−0.18
Baseline age < 75	0.64	−0.12	−0.16	−0.23	0.09^a^	−0.17
Baseline age ≥ 75	0.58	−0.21	−0.25	−0.25	0.10	−0.18
Birthyear 1900–1914	0.55	−0.17	−0.28	−0.19	0.14	−0.17
Birthyear 1915–1923	0.60	−0.22	−0.20	−0.22	0.12	−0.13
Birthyear 1924–1948	0.67	−0.12	−0.13	−0.26	0.16	−0.30

*SES* socioeconomic status, *FS* financial strain, *ISEI* international socio-economic index, *ISCED* international standard classification of education, *FAI* functional aging index

a Correlation is not significantly different from zero

**Table 3:** Phenotypic results: adding SES variables to latent growth curve parameters for FAI

Parameter	FS Estimate (SE)	ISEI Estimate (SE)	ISCED Estimate (SE)

Intercept	47.31 (0.36) **	46.63 (0.37) **	47.84 (0.34) **
Sex	2.88 (0.51) **	2.65 (0.53) **	2.91 (0.48) **
Parental SEI	−0.69 (0.30) *	−0.22 (0.32)	0.00 (0.29)
Slope 1	4.57 (0.27) **	4.10 (0.26) **	4.36 (0.24) **
Slope 2	10.11 (0.42) **	10.17 (0.46) **	9.71 (0.39) **
Intercept × SES	0.16 (0.03) **	−0.04 (0.02) *	−1.27 (0.23) **
Slope 1 × SES	0.01 (0.02)	0.00 (0.01)	−0.28 (0.14) *
Slope 2 × SES	0.02 (0.04)	0.00 (0.02)	−0.41 (0.28)

*SES* socioeconomic status, *FS* financial strain, *ISEI* international socio-economic index, *ISCED* international standard classification of education. Slope parameters estimate change over 10 years

* *p* <.05

** *p* <.01

**Table 4:** Parameter estimates for within-twin and between-twin SES variables in latent growth curve models of FAI

Effect	FS on intercept^a^ Estimate (SE)	ISEI on intercept Estimate (SE)	ISCED on intercept Estimate (SE)	ISCED on slope 1 Estimate (SE)

Full sample				
Between	0.21 (0.03) **	−0.04 (0.02) *	−1.47 (0.28) **	−0.24 (0.17)
Within	0.11 (0.03) **	−0.05 (0.02) *	−1.56 (0.35) **	−0.46 (0.24) *
MZ twins				
Between	0.25 (0.06) **	−0.08 (0.04) *	−1.60 (0.47) **	−0.24 (0.30)
Within	0.01 (0.06)	−0.08 (0.05)	−0.40 (0.63)	0.13 (0.50)
DZ twin				
Between	0.17 (0.04) **	−0.07 (0.03) *	−1.53 (0.33) **	−0.33 (0.21)
Within	0.14 (0.04) **	−0.04 (0.03)	−1.93 (0.41) **	−0.63 (0.25) *

*SES* socioeconomic status, *FS* financial strain, *ISEI* international socio-economic index, *ISCED* international standard classification of education, *MZ* monozygotic, *DZ* dizygotic

a Model corrected for parental SEI

* *p* <.05

** *p* <.01

**Table 5:** Sex differences in parameter estimates for within-twin and between-twin SES variables in latent growth curve models of FAI

Parameter	Full sample Estimate (SE)	MZ twins Estimate (SE)	DZ twins Estimate (SE)

Financial strain^a^			
Between pair FS × int. (women)	0.21 (0.02)**	0.14 (0.04)**	0.22 (0.06)**
Between pair FS × int. (correction for men)	0.00 (0.04)	0.16 (0.06)**	−0.16 (0.10)
Within pair FS × int. (women)	0.13 (0.03)**	0.10 (0.06)	0.14 (0.08)
Within pair FS × int. (correction for men)	−0.06 (0.05)	−0.21 (0.09)*	−0.06 (0.12)
Occupation			
Between pair ISEI × int (women)	−0.04 (0.03)	−0.03 (0.05)	−0.05 (0.03)
Between pair ISEI × int (correction for men)	0.01 (0.03)	−0.01 (0.06)	0.02 (0.04)
Within pair ISEI × int. (women)	−0.05 (0.03)	−0.08 (0.06)	−0.06 (0.04)
Within pair ISEI × int. (correction for men)	0.02 (0.05)	0.05 (0.08)	0.04 (0.05)
Education			
Between pair ISCED × int. (women)	−1.24 (0.28)**	−0.43 (0.44)	−1.13 (0.46)*
Betw pair ISCED × int. (correction for men)	0.15 (0.40)	−1.30 (0.63)*	1.07 (0.66)
Within pair ISCED × int (women)	−0.76 (0.51)	−0.61 (0.87)	−1.30 (0.77)
Within pair ISCED × int (correction for men)	−1.32 (0.72)	0.30 (1.30)	−0.78 (1.05)

*SES* socioeconomic status, *FS* financial strain, *ISEI* international socio-economic index, *ISCED* international standard classification of education, *MZ* monozygotic, *DZ* dizygotic, *c. for men* correction for men

a Model corrected for parental SEI

* *p* <.05

** *p* <.01

## Data Availability

This research used third-party data and materials accessed through the IGEMS consortium. The following are a list of participating studies with links to how to access or to request data from each study: SATSA data are publicly available through National Archive of Computerized Data on Aging (NACDA). See [https://doi.org/10.3886/ICPSR03843.v2](https://doi.org/10.3886/ICPSR03843.v2) Investigators may apply for OCTO-Twin data through University of Gothenburg. See [https://www.gu.se/en/research/the-octo-twin-study-origins-of-variance-in-the-old-old](https://www.gu.se/en/research/the-octo-twin-study-origins-of-the-old-old) Investigators may apply for access to data from all three studies through National E-infrastructure for Aging Research (NEAR). See [https://www.near-aging.se/studies-included/application/](https://www.near-aging.se/studies-included/application).
