# Supplementary material for: Co-twin Control Analyses Reveal Genetic Contributions to SES Influences in Mean Level and Longitudinal Change in Physical Aging
Source: Behav Genet. Author manuscript; Available in PMC 2026 Apr 7. (PMC13053737; doi:10.1007/s10519-026-10256-2)
Supplement: Supplemental Material [file NIHMS2155455-supplement-Supplemental_Material.pdf]

Finkel, D., Finch, B. K., Gatz, M., Karlsson, I. K., Reynolds, C. A., Mosing, M., Nimmagadda, S., & Ericsson, M. (2026). Co-twin Control Analyses Reveal Genetic Contributions to SES Influences in Mean Level and Longitudinal Change in Physical Aging. *Behavior Genetics*, 1-13.

https://doi.org/10.1007/s10519-026-10256-2

Supplemental Material

Supplemental Table 1. Sample demographics for men and women

Variable	Men	Women
N individuals	648	721
N individuals with parental SEI	612	681
N of MZ/N of DZ pairs	67/176	75/195
MZ/DZ pairs with parental SEI	58/168	65/188
Age Range at Baseline (yrs)	45 - 92	42 – 93
Mean Age at Baseline (SD)	71.60 (10.18)	71.97 (10.82)
Mean Waves Participation (SD)	3.20 (1.95)	3.34 (2.04)
Mean Length Follow-up (SD)	9.62 (6.98)	10.73 (7.55)**
Mean ISCED (SD)	1.95 (1.49)	1.67 (1.26)**
MZ twin correlation	.60	.49
DZ twin correlation	.40	.48
Mean Financial Strain (SD)	47.57 (11.09)	48.19 (10.63)
MZ twin correlation	.43	.52
DZ twin correlation	.11	.11
Mean ISEI (SD)	38.82 (20.04)	33.87 (18.27)**
MZ twin correlation	.51	.58
DZ twin correlation	.29	.31
Mean Parental SEI (SD)[a]	2.06 (0.86)	2.17 (0.93)*

Note: MZ = monozygotic twin, DZ = dizygotic twin, ISCED = International Standard Classification of Education, ISEI = International Socio-Economic Index, SEI = Socio-Economic Index

* mean difference between men and women is significant at $p < .05$

** mean difference between men and women is significant at $p < .01$

Supplemental Table 2. Adding within twin pair and between twin pair SES variables to latent growth curve parameters for FAI

Parameter	Full Sample<br>Estimate (SE)	MZ twins<br>Estimate (SE)	DZ twins<br>Estimate (SE)
Financial Strain			
Intercept	47.29 (0.36)**	49.38 (0.66)**	46.64 (0.42)**
Sex	2.96 (0.51)**	3.28 (1.03)**	2.93 (0.58)**
Parental SEI	-0.67 (0.30)*	-0.73 (0.52)	-0.74 (0.35)*
Between pair FS x intercept	0.21 (0.03)**	0.25 (0.06)**	0.17 (0.04)**
Within pair FS x intercept	0.11 (0.03)**	0.01 (0.06)	0.14 (0.04)**
Slope 1	4.67 (0.26)**	5.13 (0.46)**	4.57 (0.30)**
Slope 2	10.13 (0.42)**	9.69 (0.73)**	10.10 (0.51)**
Occupation			
Intercept	46.69 (0.37)**	50.82 (0.75)**	49.99 (0.67)**
Sex	2.52 (0.52)**	2.92 (1.30)*	2.90 (0.95)**
Between pair ISEI x intercept	-0.04 (0.02)*	-0.08 (0.04)*	-0.07 (0.03)*
Within pair ISEI x intercept	-0.05 (0.02)*	-0.08 (0.05)	-0.04 (0.03)
Slope 1	4.08 (0.25)**	5.14 (0.46)**	5.28 (0.35)**
Slope 2	10.08 (0.43)**	10.55 (1.03)**	11.64 (1.02)**
Education			
Intercept	48.66 (0.36)**	52.46 (0.65)**	47.59 (0.41)**
Sex	2.92 (0.52)**	3.12 (1.08)**	2.79 (0.59)**
Between pair ISCED x intercept	-1.47 (0.28)**	-1.60 (0.47)**	-1.53 (0.33)**
Within pair ISCED x intercept	-1.56 (0.35)**	-0.40 (0.62)	-1.93 (0.41)**
Slope 1	4.54 (0.24)**	5.58 (0.44)**	4.28 (0.29)**
Between pair ISCED x slope 1	-0.24 (0.17)**	-0.24 (0.29)	-0.33 (0.21)
Within pair ISCED x slope 1	-0.46 (0.24)	0.13 (0.49)	-0.63 (0.25)*
Slope 2	10.58 (0.47)**	10.63 (0.92)**	10.49 (0.55)**

* $p < .05$; ** $p < .01$

Supplemental Table 3. Adding within twin pair and between twin pair SES variables to latent growth curve parameters for FAI, stratified by sex. Primary results are for women, parameters for men indicate the correction (or difference in value) for men.

Parameter	Full Sample<br>Estimate (SE)	MZ twins<br>Estimate (SE)	DZ twins<br>Estimate (SE)
Financial Strain			
Intercept (women)	49.41 (0.31)**	52.40 (0.60)**	51.03 (0.67)**
Intercept (correction for men)	-3.48 (0.32)**	-3.96 (0.53)**	-2.87 (0.87)**
Parental SEI	-0.38 (0.18)*	-0.47 (0.31)	-0.31 (0.44)
Between pair FS x int. (women)	0.21 (0.02)**	0.14 (0.04)**	0.22 (0.06)**
Between pair FS x int. (c. for men)	0.00 (0.04)	0.16 (0.06)**	-0.16 (0.10)
Within pair FS x int. (women)	0.13 (0.03)**	0.10 (0.06)	0.14 (0.08)
Within pair FS x int. (c. for men)	-0.06 (0.05)	-0.21 (0.09)*	-0.06 (0.12)
Slope 1	4.70 (0.30)**	6.20 (0.51)**	5.68 (0.33)**
Slope 2	7.33 (0.38)**	6.22 (0.65)**	9.31 (0.68)**
Occupation			
Intercept	47.89 (0.47)**	50.81 (0.95)**	47.06 (0.54)**
Intercept (correction for men)	-2.65 (0.54)**	-3.59 (1.12)**	-2.51 (0.61)**
Between pair ISEI x int (women)	-0.04 (0.03)	-0.03 (0.05)	-0.05 (0.03)
Between pair ISEI x int (c. for men)	0.01 (0.03)	-0.01 (0.06)	0.02 (0.04)
Within pair ISEI x int. (women)	-0.05 (.03)	-0.08 (0.06)	-0.06 (0.04)
Within pair ISEI x int. (c. for men)	0.02 (0.05)	0.05 (0.08)	0.04 (0.05)
Slope 1	4.07 (0.26)**	4.65 (0.47)**	3.87 (0.31)**
Slope 2	10.20 (0.45)**	8.83 (0.77)**	10.46 (0.56)**
Education			
Intercept (women)	49.51 (0.36)**	52.10 (0.64)**	51.15 (0.56)**
Intercept (correction for men)	-2.95 (0.48)**	-3.56 (0.80)**	-2.85 (0.74)**
Between pair ISCED x int. (women)	-1.24 (0.28)**	-0.43 (0.44)	-1.13 (0.46)*
Betw pair ISCED x int. (c. for men)	0.15 (0.40)	-1.30 (0.63)*	1.07 (0.66)
Within pair ISCED x int (women)	-0.76 (0.51)	-0.61 (0.87)	-1.30 (0.77)
Within pair ISCED x int (c. for men)	-1.32 (0.72)	0.30 (1.30)	-0.78 (1.05)
Slope 1	4.54 (0.22)**	5.19 (0.38)**	4.91 (0.27)**
Slope 2	9.33 (0.29)**	8.38 (0.52)**	8.69 (0.41)**

* $p < .05$; ** $p < .01$

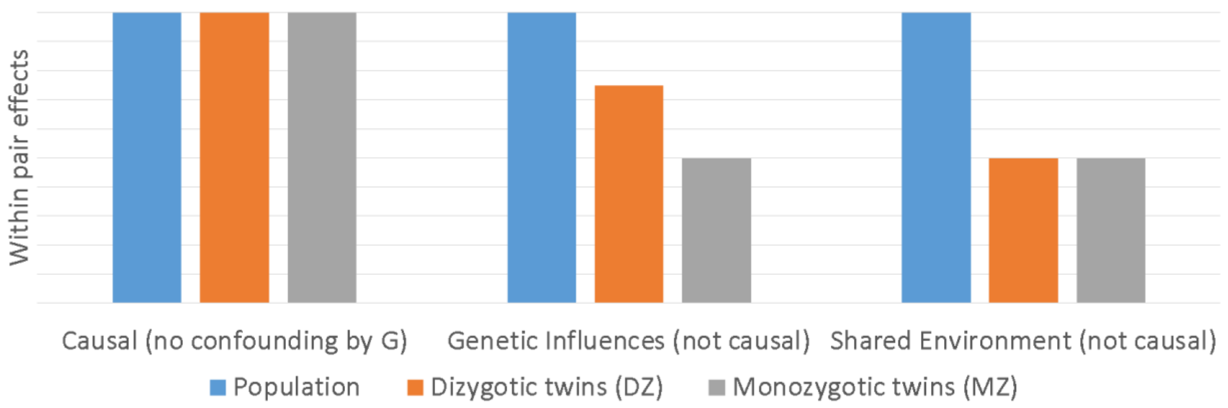

Supplemental Figure 1: Expected results for total sample, DZ twin, and MZ twins under three different models of the mechanism of the relationship between SES and FAI.

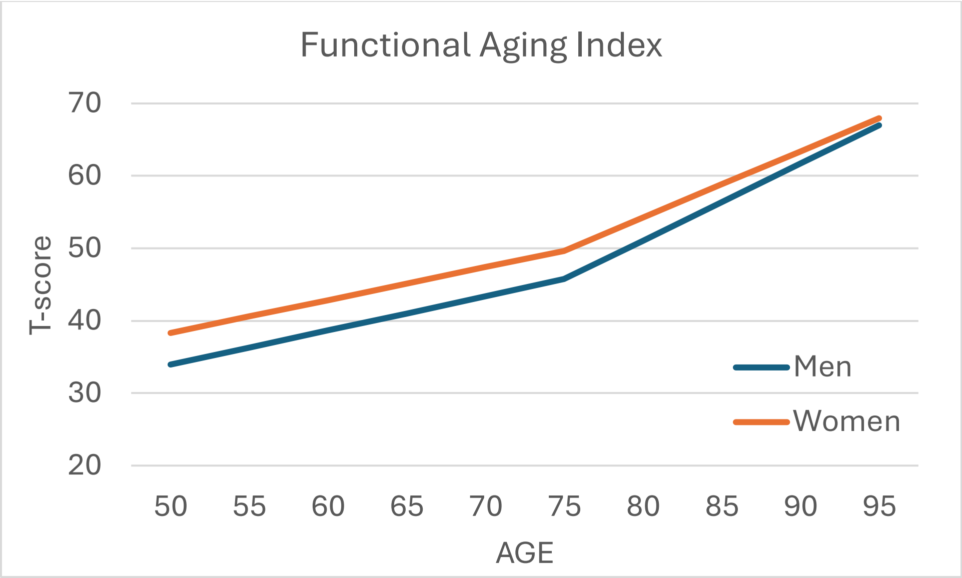

Supplemental Figure 2. Estimated longitudinal trajectories for FAI for men and women.
